# Los análisis de orina en el diagnóstico de las gammapatías monoclonales: puntos de corte

**DOI:** 10.1515/almed-2024-0184

**Published:** 2024-12-10

**Authors:** Mònica Vidal-Pla, Elisa Nuez-Zaragoza, Indira Bhambi, Vicente Aguadero

**Affiliations:** Laboratorio Clínico, Servicio de Análisis Clínicos, Hospital Parc Taulí University, Institut d’Investigació i Innovació Parc Taulí I3PT, Universitat Autònoma de Barcelona, Sabadell, España; Servicio de Laboratorio, Hospital Sant Joan Despí Moisès Broggi, CLILAB Diagnòstics, Barcelona, España

**Keywords:** puntos de corte, gammapatía monoclonal, componente monoclonal, análisis de orina

## Abstract

**Objetivos:**

Las pruebas de laboratorio resultan cruciales en el diagnóstico de las gammapatías monoclonales. La última actualización de la guía del Grupo Internacional de Trabajo sobre el Mieloma (IMWG) para el diagnóstico de gammapatías monoclonales ha suscitado cierto debate sobre la pertinencia de realizar análisis de orina en el diagnóstico de estas patologías.

**Métodos:**

Realizamos un estudio retrospectivo con datos de 132 pacientes con detección reciente de componente monoclonal (CM) en suero. Se dividió a los pacientes en dos grupos, en función de la presencia o ausencia del componente monoclonal (CM) en la orina. A continuación, se recogieron diversas variables para analizar posibles diferencias entre los grupos.

**Resultados:**

El objetivo del presente estudio era establecer un punto de corte para la concentración de CM en suero por debajo del cual no fuese necesario realizar un análisis de orina en el diagnóstico inicial en el laboratorio. Los resultados muestran que, cuando la concentración de CM en suero es ≤3,5 g/L y la eGFR es >30 mL/min/1,73m^2^ (sensibilidad (S): 100 %, especifidad (Sp): 49 %, valor predictivo negativo (VPN): 100 %), la probabilidad de hallar CM en orina es mínima. Los pacientes con CM con cadena pesada alfa tienen mayor probabilidad de presentar CM en la orina. De este modo, cuando la cadena pesada el CM es gamma o mu, el punto de corte en suero se puede elevar a ≤4,9 g/L (S: 97 %, Sp: 52 %, VPN: 98 %).

**Conclusiones:**

El empleo de estos dos puntos de corte en la detección inicial de un CM podría evitar la realización innecesaria de un número considerable de análisis de orina, optimizando así el consumo de recursos analíticos y sanitarios.

## Introducción

Las pruebas de laboratorio resultan cruciales en el diagnóstico de las gammapatías monoclonales. La última actualización de la guía del Grupo Internacional de Trabajo sobre el Mieloma (IMWG) para el diagnóstico de gammapatías monoclonales ha suscitado cierto debate sobre la pertinencia de realizar análisis de orina en el diagnóstico de estas patologías [1–3].

En la guía 2014 del IMWG, únicamente se considera relevante el análisis de orina para el diagnóstico de la amiloidosis, la gammapatía monoclonal de cadenas ligeras de significado incierto y el mieloma latente. Los criterios diagnósticos para la gammapatía monoclonal de significado incierto (MGUS, por sus siglas en inglés) son: componente monoclonal (CM) en suero <30 g/L, células plasmáticas de médula ósea clonales <10 % y ausencia de síntomas CRAB. La concentración de CM en orina no se incluye como criterio, ya que la presencia de CM en orina no cambia el diagnóstico, aunque los clínicos la solicitan muy frecuentemente [1].

Se recomienda realizar el análisis en orina de 24h. Sin embargo, a algunos pacientes les puede resultar difícil tomar la muestra, lo que puede derivar en resultados imprecisos. La electroforesis de proteínas en orina (EFPO) y la inmunofijación en orina son técnicas que requieren mucho tiempo, así como un paso previo de concentración, no estando además completamente automatizadas. Así mismo, la inmunofijación en orina requiere de una interpretación manual por parte de un profesional experimentado [4, 5].

Por todo ello, diseñamos un estudio para probar la hipótesis de que, cuando un paciente presenta una concentración baja de CM en suero en la primera prueba analítica para la detección de una gammapatía, la probabilidad de hallar CM en la orina es también baja. El objetivo principal de este estudio es establecer un punto de corte para el CM en suero por debajo de la cual la probabilidad de hallar CM en orina sea muy baja, por lo que un análisis de orina no sería necesario. Por otro lado, analizamos la capacidad de otros parámetros para predecir la ausencia de CM en orina.

## Materiales y métodos

Se llevó a cabo un estudio retrospectivo en pacientes con presencia de CM de diagnóstico reciente. Se excluyó a aquellos pacientes con paraproteínas biclonales, paraproteínas monoclonales de cadena ligera y amiloidosis. Como criterio secundarios de inclusión, se incluyó a pacientes a los que que se les había realizado un análisis EFPO en los dos meses posteriores al hallazgo de CM en sangre.

El presente estudio retrospectivo observacional fue aprobado por el Comité de Ética de Investigación en Productos Medicinales del Hospital (Código 20235011).

La población de estudio estaba compuesta por pacientes ingresados en nuestro hospital o derivados al mismo desde consultas externas. Los datos clínicos de los pacientes se extrajeron del sistema de gestión de información del hospital, para asegurarnos de que cumplían los criterios de inclusión. los datos recogidos fueron la edad, el sexo, la concentración de CM en suero (g/L), el tipo de cadena pesada (IgG, IgA, IgM), el tipo de cadena ligera (kappa o lambda) y las concentraciones séricas totales de las tres inmunoglobulinas (IgG, IgA y IgM). Asimismo, se registró si el CM estaba también presente en la orina, así como la concentración total de proteínas en orina y la estimación de la tasa de filtración glomerular (TFGe), calculada mediante la ecuación de la Chronic Kidney Disease Epidemiology Collaboration 2009 (CKD-EPI). Cada paciente fue asignado a uno de los siguientes dos grupos, en función de la presencia o ausencia de CM en la orina: grupo A, pacientes sin CM en la orina; grupo B: pacientes con CM en la orina (se consideró positiva la detección en orina tanto de inmunoglobulinas intactas monoclonales como de cadenas ligeras libres monoclonales).

El proteinograma sérico se realizó mediante electroforesis capilar utilizando V8^®^ Nexus (Helena Biosciences Europe, Gateshead, Reino Unido). La EFPO se realizó mediante electroforesis en gel utilizando SAS3 (Helena Biosciences Europe, Gateshead, Reino Unido). La presencia de picos sugestivos en los proteinogramas de suero y orina se investigó mediante inmunofijación en SAS3 para su confirmación y caracterización. Tras la EFPO, se realizó una inmunofijación en todas las muestras de orina. La concentración de CM en suero se midió mediante espectrometría UV, mientras mientras que en orina se midió meidante densiometría. En ambos casos, se empleó un método perpendicular para la integración.

La concentración de inmunoglobulinas se determinó con un analizador Optilite^®^ (The Binding Site Group Ltd., Birmingham, Reino Unido) mediante turbidimetría. Las proteínas en orina se analizaron mediante turbidimetría con el módulo c702 de la plataforma analítica Cobas 8000 (Roche Diagnostics Mannheim, Alemania).

Los análisis estadísticos se realizaron con el programa SPSS Statistics versión 25.0 (IBM; Armonk, NY, EE.UU.). La significación estadística se estableció en p< 0,05. Se aplicó la prueba de Kolmogorov–Smirnov para comprobar la normalidad de la distribución de las variables cuantitativas, mientras que, para comparar las frecuencias en los dos grupos, se aplicaron las pruebas de Chi cuadrado y la prueba de Fisher.

Por otro lado, con la prueba U de Mann–Whitney se analizaron las diferencias en las medias de las variables cuantitativas obtenidas en los grupos A y B. Cuando se observaron diferencias estadísticamente significativas en la media de los dos grupos, se construyeron curvas ROC, estableciendo la presencia de CM en la orina como variable de estado.

## Resultados

Entre febrero de 2019 y marzo de 2022, se registraron datos de 132 pacientes correspondientes a 132 muestras (Tablas 1 y 2). Tras realizar el análisis de datos, se observó una relación estadísticamente significativa y directamente proporcional entre la presencia de CM en orina, la concentración de CM en suero y las proteínas totales en orina. Por otro lado, el estudio reveló una relación inversamente proporcional entre la presencia de CM en orina y la eGFR y la concentración de IgM. No se observaron diferencias estadísticamente significativas en la distribución de las edades de los pacientes o en la concentración total de IgG e IgA (Tabla 1).

**Tabla 1:** Datos demográficos y características clínicas de los pacientes.

	Mediana (RIC) del Grupo A	Mediana (RIC) del Grupo B	p	Área bajo la curva ROC (IC95 %)
Edad, años	71 (20)	75 (20)	0,272	^a^
CM en suero, g/L	3,28 (4,51)	15,1 (29,1)	**<0,001**	0,794 (0,706–0,883)
PT en orina, g/L	0,10 (0,14)	0,45 (1,05)	**<0,001**	0,761 (0,663–0,857)
eGFR, mL/min/1,73m^2^	78 (26)	59 (54)	**0,001**	0,691 (0,585–0,797)
IgG, g/L	12,22 (6,08)	11,69 (19,84)	0,516	^a^
IgA, g/L	2,54 (2,28)	1,39 (5,99)	0,088	^a^
IgM, g/L	0,995 (1,08)	0,32 (0,81)	**<0,001**	0,727 (0,609–0,845)

Los valores p estadísticamente significativos están resaltados en negrita. ^a^No se construyó una curva ROC, dado que la variable no eraestadísticamente significativa. IC, intervalo de confianza; eGFR, Tasa de filtración glomerular estimada; IgA, Inmunoglobulina A; IgG, inmunoglobulina G; IgM, inmunoglobulina M; RIC, rango intercuartílico; ROC, características operativas del receptor; PT, proteínas totales.

Los análisis de variables cualitativas no revelaron diferencias estadísticamente significativas en la distribución por sexo y tipo de cadena ligera entre los dos grupos. Sin embargo, sí se observaron diferencias estadísticamente significativas en la distribución de cadena ligera y pesada. Esto nos llevó a analizar la distribución de los CM de tipo IgG, IgA, e IgM en los dos grupos, observando una mayor probabilidad de presentar CM en la orina cuando el isotipo era alfa, y una menor probabilidad cuando el isotipo era gamma (Tabla 2).

**Tabla 2:** Distribución de los pacientes según el sexo y el tipo de cadena pesada y ligera del CM en suero.

		Grupo A Nº de pacientes, %	Grupo B Nº de pacientes, %	TOTAL	p
Sexo	Hombre	54 (57 %)	22 (59 %)	76	0,471
	Mujer	41 (43 %)	15 (41 %)	56	
Tipo de cadena pesada	G	73 (77 %)	22 (59 %)	95	**0,032**
	A	10 (11 %)	11 (30 %)	21	
	M	12 (12 %)	4 (11 %)	16	
Tipo de cadena ligera	K	58 (61 %)	20 (54 %)	78	0,294
	L	37 (39 %)	17 (46 %)	54	
Total		95 (72 %)	37 (28 %)	132	

Los valores p estadísticamente significativos están resaltados en negrita. A, alfa; G, gamma; K, kappa; L, lambda; M, mu.

Para todas las variables con un valor p<0,05, se construyeron curvas ROC para identificar puntos de corte con valores predictivos óptimos para la ausencia de CM en orina (Material Suplementario). Al establecer como punto de corte una concentración de CM en suero ≤3,5 g/L, obtuvimos una sensibilidad del 86 % y una especificidad del 53 %, con un valor predictivo negativo de 91 % (VPN). Al revisar las historias de los pacientes con una concentración de CM en suero ≤3,5 g/L y presencia de CM en la orina, observamos que dichos pacientes presentaban niveles muy bajos de eGFR. Por ello, añadimos un eGFR >30 mL/min/1,73 m^2^ al punto de corte anterior, logrando aumentar la sensibilidad al 100 %, con una especificidad del 49 % y un VPN del 100 % para la presencia de CM en la orina (Tabla 3). Este punto de corte podría haber evitado la realización de 47 análisis de orina (35,6 %) en nuestra población de estudio.

**Tabla 3:** Sensibilidades, especificidades y valores predictivos negativos de los valores de referencia propuestos.

	n	S	Sp	NPV
CM en suero ≤3,5 g/L	54	86 %	53 %	91 %
CM en suero ≤3,5 g/L + eGFR >30 mL/m/1,73	47	100 %	49 %	100 %
CM IgG o IgM en suero ≤4,9 g/L + eGFR >30 mL/m/1,73	56	97 %	52 %	98 %

eGFR, tasa de filtración glomerular estimada; IgM, inmunoglobulina M; IgG, inmunoglobulina G; n, número de sujetos; VPN, valor predictivo negativo; S, sensibilidad; Sp, especificidad.

Asimismo, observamos una relación estadísticamente significativa entre la presencia de CM en la orina y el isotipo de cadena pesada de CM. La probabilidad de presentar CM en la orina fue mayor en los pacientes con CM de tipo IgA (Tabla 2). De este modo, cuando la cadena pesada del CM era gamma o mu, nos permitió aumentar el aumentar el punto de corte en suero a ≤4,9 g/L y manteniendo el la eGFR >30 mL/min/1,73m^2^ se obtuvieron una sensibilidad (97 %), especifidad (52 %), y VPN (98 %) similares (Tabla 3). En este caso, se habrían evitado 56 análisis de orina.

## Discusión

Los análisis de orina mediante EFPO e inmunofijación constituyen una parte esencial del estudio completo tras detectar la presencia de una gammapatía monoclonal en la práctica clínica. No obstante, existen ciertas discrepancias en las guías clínicas en lo relativo a la pertinencia de realizar dichas pruebas, ya que estas pueden no ser necesarias en algunos casos, dado que las técnicas séricas pueden aportar suficiente información para el diagnóstico inicial de dichas patologías. El análisis de orina no aparece en la guía 2014 del IMWG como criterio diagnóstico para la MGUS [1]. Por otro lado, en las nuevas recomendaciones de consenso para el estudio de las gammapatías monoclonales de la Sociedad Española de Medicina de Laboratorio y la Sociedad Española de Hematología y Hemoterapia, no se recomienda realizar análisis de orina en el cribado inicial de gammapatía monoclonal, aunque sí se recomienda realizarlos una vez se ha detectado CM en sangre, con el fin de completar el estudio [6].

Diversos autores han analizado el papel de los análisis de orina en el diagnóstico de las gammapatías monoclonales. Así, Beetham y col. evaluaron la necesidad de incluir análisis de orina en el algoritmo diagnóstico de las gammapatías monoclonales. Sus resultados mostraron que solo se habría pasado por alto uno de cada 105 diagnósticos si se hubieran realizado únicamente análisis de sangre (electroforesis del suero, inmunofijación del suero y concentración de cadenas ligeras libres en suero) [7]. No obstante, los autores concluyeron que no se podían eliminar los análisis de orina del algoritmo de cribado de gammapatía monoclonal. Katzmann y col. publicaron un estudio similar en el que solo dos de cada 425 pacientes con gammapatías monoclonales habrían recibido un diagnóstico erróneo si únicamente se les hubieran realizado pruebas séricas. Los autores de dicho estudio concluyeron que, debido al pequeño incremento de la sensibilidad aportado por los análisis de orina, no sería necesario realizarlos en el estudio inicial, pudiendo estos ser solicitados posteriormente y de manera más selectiva [8, 9].

A diferencia de otros estudios, proponemos un punto de corte a partir del cual se podría obviar la realización de análisis de orina, mostrando un VPN muy elevado (100 % para la concentración de CM ≤3,5 g/L y 98 % para concentraciones de CM IgG o IgM ≤4,9 g/L).

Una de las ventajas de incorporar estos valores de corte en el algoritmo del laboratorio sería que no sería necesario obtener muestras de orina de aquellos pacientes que cumplieran los criterios oportunos, ya que estos criterios se basan en parámetros determinados en suero (CM en suero y creatinina en suero para la ecuación CKD-EPI de eGFR). Los valores de referencia (puntos de corte) propuestos también serían aplicables a pacientes con disfunción renal con eGFR >30 mL/min/1,73m^2^.

Cabe señalar que nuestro estudio se realizó en un grupo específico de pacientes (primera vez que se detecta CM en suero), por lo que sería necesario realizar más investigaciones para determinar si estos valores de corte mostrarían buenos valores predictivos en otros grupos de pacientes (p.ej. pacientes con la presencia de dos tipos de CM, paraproteína de cadena ligera, pacientes en tratamiento, y pacientes en remisión).

Como resultado de este estudio podemos concluir que, en los pacientes con hallazgo inicial de CM en suero, los análisis de orina no serían necesarios cuando la concentración de CM en suero es ≤3,5 g/L y la eGFR es >30 mL/min/1,73m^2^. En este contexto, se podría evitar un número significativo de análisis de orina, optimizando de este modo el uso de recursos clínicos y analíticos.

## Supplementary Material

Supplementary Material Details

## Material Suplementario

Este artículo incluye material suplementario (https://doi.org/10.1515/almed-2024-0184).
