# Supplementary figures and images for: Los análisis de orina en el diagnóstico de las gammapatías monoclonales: puntos de corte
Source: Adv Lab Med. 2024 Dec 10;5(4):443–6. [Article in Spanish] doi: 10.1515/almed-2024-0184 (PMC11661538; doi:10.1515/almed-2024-0184)

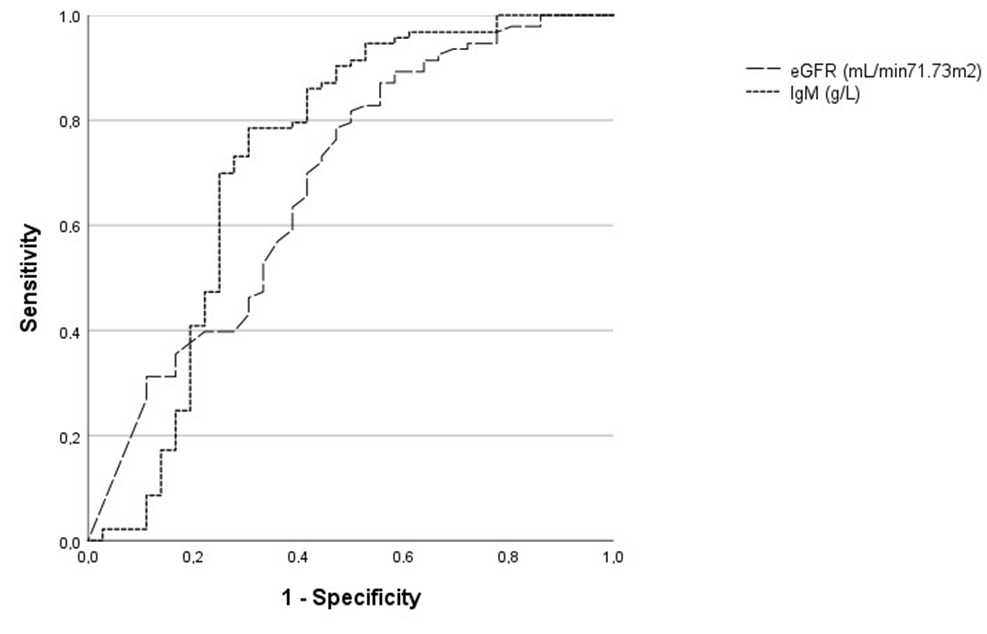
**Curvas ROC**

**Sensibilidad**

**Sensibilidad**

Supplement: Supplementary file 1 — Supplementary Material Details [file j_almed-2024-0184_suppl_001.docx]
